# Ab initio calculation for electronic structure and optical property of tungsten carbide in a TiCN-based cermet for solar thermal applications

**DOI:** 10.1038/s41598-023-36337-4

**Published:** 2023-06-09

**Authors:** Shota Hayakawa, Toshiharu Chono, Kosuke Watanabe, Shoya Kawano, Kazuma Nakamura, Koji Miyazaki

**Affiliations:** 1 Graduate School of Engineering, Kyushu Institute of Technology, 1-1 Sensui-cho, Tobata-ku, Kitakyushu, Fukuoka 804-8550 Japan; 2 Integrated Research Center for Energy and Environment Advanced Technology, Kyushu Institute of Technology, 1-1 Sensui-cho, Tobata-ku, Kitakyushu, Fukuoka 804-8550 Japan; 3 Graduate School of Engineering, Kyushu University, 744 Motooka, Nishi-ku, Fukuoka, 819-0395 Fukuoka Japan

**Keywords:** Electronic structure, Nanophotonics and plasmonics, Electronic properties and materials, Electronic properties and materials, Nanophotonics and plasmonics, Thermoelectric devices and materials

## Abstract

We present an ab initio calculation to understand electronic structures and optical properties of a tungsten carbide WC being a major component of a TiCN-based cermet. The TiCN-based cermet is widely used as a cutting tool, and is discarded as usual after use. On the other hand, cermet itself is also a famous ingredient of a solar absorption film. We found that the WC has a fairly low-energy plasma excitation $\sim$ 0.6 eV (2 $\upmu$m) and therefore can be a good constituent of a solar selective absorber. The evaluated figure of merit for photothermal conversion is prominently high compared to those of the other materials included in the TiCN-based cermet. The imaginary part of the dielectric function is considerably small around the zero point of the real part of the dielectric function, corresponding to the plasma excitation energy. Therefore, a clear plasma edge appeared, ensuring the high performance of the WC as the solar absorber. This is a fascinating aspect, because the wasted TiCN-based cermet cutting tool can be recycled as the solar absorption film after proper treatments and modifications.

## Introduction

The replacement of fossil fuels to renewable energy sources have been intensively investigated in recent years. Solar energy has been considered as promising alternative to solve global energy issues due to its abundance^1^. Exploring sustainable and eco-friendly technologies has been considered significant to achieve the practical use of harvesting solar energy. Photovoltaic conversion is most widespread technology to directly generate electric power from solar power. On the other hand, cermet-based solar absorbers have been also commercialized to obtain thermal energy from the sunlight^2^. Concentrated solar power plant is one of proven technologies, which generates the electricity by a steam turbine. Thermal energy can be storage to generate the electricity when the sun is no longer shining. Thermal energy storage is significantly cheaper than other energy storage system (e.g. batteries)^3^. However, three limitations of an artificial solar absorber have been identified for improving the solar thermal system^4^. First, solar absorbers with high efficiency are made through complicated designs using sub-wavelength structured metamaterials. Second, traditional preparation methods require expensive vacuum-based deposition equipment and high-purity targets to create a multilayered surface. Third, thermal stability is insufficient to maintain their spectral selective absorptance during long-term high-temperature operation.

**Figure 1:**
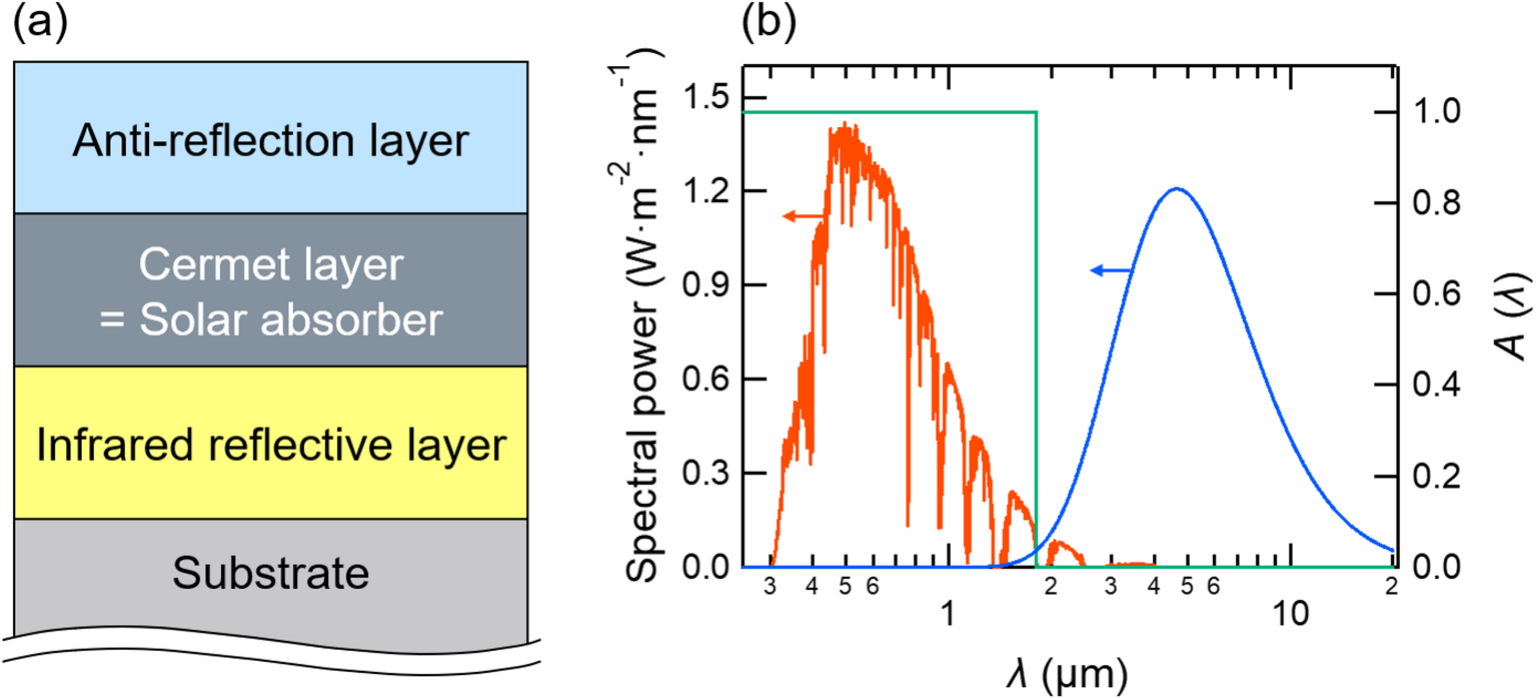
(**a**) A schematic figure of a cermet-based solar absorber consisting of an anti-reflection layer, a cermet layer, and an infrared reflective layer. (**b**) A schematic figure to see the optical performance required as the solar absorber. A red solid curve describes the spectral power of the sunlight ranging from 0.3 to 2.0 $\upmu$m, and a blue solid curve is the spectral power of the thermal irradiation of the black body. Thus, 2 $\upmu$m is a cutoff wavelength ensuring high solar absorptivity and low thermal emissivity. Ideally, a material with an absorptivity spectrum $A(\lambda )$ described with a green solid line is preferable as the solar absorber.

Cermet is a composite of metal and ceramic with hardness, thermal stability, and anti-oxidation properties. A typical solar absorber is shown in Fig. 1a^1,2^. The cermet-based solar absorber consists of a cermet layer as absorber with an anti-reflection layer on the top and an infrared reflector at the bottom. The solar selective absorber is an important role to achieve the high performance as a solar absorber. The blackbody emissive power significantly increases in high temperature, resulting in large radiative heat loss from the absorber. An ideal solar selective absorber should have high solar absorptivity and low thermal emissivity as described with a green line in Fig. 1b with 2.0 $\upmu$m cutoff wavelength^5^. The cermet-based absorbers have been well investigated with oxides with metal particles. The dielectric function of the composite is controlled by increasing metal volume fraction to decrease the frequencies of absorption peaks analyzed by Bruggeman approximation^3^.

Materials with low-plasma excitation is preferable for a solar selective absorber. For further improvement of the performance, the multi-layered solar selective absorbers have been investigated using optical interference. The vacuum deposition process with high production costs is usually needed to precisely control the film thickness^2,3^. Here, we intend to find materials applicable to a solar selective absorber with low production costs. A TiCN cermet including various metals, carbide, and nitride is generally used for hard machining tools due to their toughness mentioned above^6^. After the service time by wear of the machining tools, the fine powder of cermet is made as industrial waste disposal. The production costs can be significantly reduced by using wasted materials for solar energy utilization.

In the present study, to discuss the performance of the TiCN-based cermet as a solar selective absorber, we perform ab initio optical analyses for this cermet, focusing on its major components of W, WC, TiC, and TiN. Systematic investigations of ab initio optical calculations have been performed for TiN and TiC in a face-centered cubic (fcc) structure^7,8^ and transition metals including W in a body-centered cubic (bcc) structure^9^, but no such calculations have been performed for WC in a hexagonal closed pack (hcp) structure. We will show that the WC has considerably sharp plasma edge around 0.6 eV (2 $\upmu$m), which is a highly desirable aspect for the solar absorber. We will discuss the microscopic origin of this low-energy plasma excitation in terms of an ab initio dielectric analysis.

The rest of the paper is organized as follows: In “Materials and methods” section, we specify major components of a TiCN-based cermet to be analyzed. Based on the X-ray diffraction (XRD) pattern analysis, we chose four materials W, WC, TiC, and TiN. We also describe methodological details for ab initio band-structure and optical-response calculations. “Results and discussions” sectiondescribes computational results on the electronic structure, reflectivity spectra, and dielectric functions. We also estimate the figure of merit for photothermal conversion of the four materials. Finally, we describe in “Conclusion” section summary of the paper.

## Materials and methods

### TiCN-based cermet

**Figure 2:**
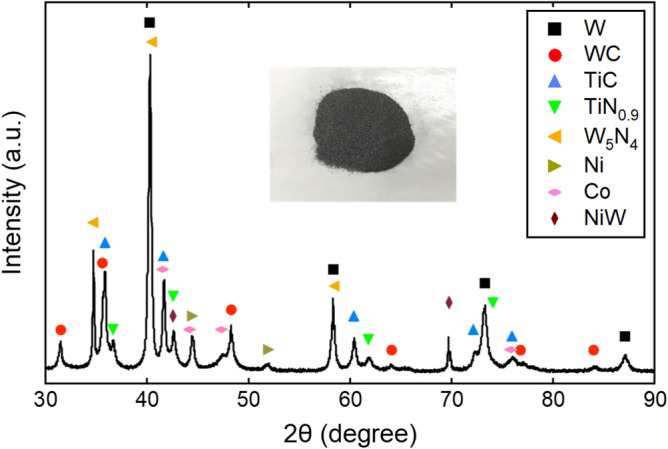
Typical XRD patterns for the TiCN-based cermet as the waste material and the crystal phase identified from the powder-diffraction-file database in the International Centre for Diffraction Data. We see that it contain various components such as transition and rare metals and their carbides and nitrides. In the present study, we focus on major four components W, WC, TiC, and TiN.

In this section, we describe a component analysis of the scrapped TiCN cermet. It is well known that the cermet consists of various metal and compounds. Our target TiCN-based cermet also contains many components, and each material can individually contribute optical property of the cermet in total. We show in the inset of Fig. 2 the TiCN cermet of the waste material^10^ to be analyzed. It is black in the visual range, so it can absorb visible light. To specify major components of the TiCN-based cermet, powder XRD analysis of the cermet powder was carried out by a SmartLab diffractometer (Rigaku Co., Ltd.) with Cu K$\alpha$ radiation ($\lambda$ = 1.5418 Å) at the scanning rate of 10.4$^\circ$/min for the 2$\theta$ value of 30 to 90$^\circ$. The result is shown in Fig. 2. We see that TiC and TiN can clearly be main components, but the present cermet also contain more major ingredients such as tungsten W and tungsten carbide WC. In the present study, therefore, we chose top major four materials WC, W, TiC, and TiN, and calculate their electronic structures and optical properties. We note in passing that all these materials have very high melting temperature near 3000 K (Table 1) and are therefore well tolerated to working temperature range in the solar absorber.

**Table 1:** Calculated and experimental lattice parameters of WC, W, TiC, and TiN, where the WC is an hcp structure, and the W is a bcc structure, and the TiC and TiN are an fcc structure.

	WC (hcp)		W (bcc)		TiC (fcc)		TiN (fcc)	
	Theory	Expt.	Theory	Expt.	Theory	Expt.	Theory	Expt.
*a*	2.922	2.91 (Ref. ^11^)	3.184	3.165 (Ref. ^12^)	4.333	4.328 (Ref. ^13^)	4.247	4.250 (Ref. ^13^)
*c*	2.847	2.84 (Ref. ^11^)	–	–	–	–	–	–
$T_{\textrm{m}}$	3028 (Ref. ^14^)		3149 (Ref. ^15^)		2792 (Ref. ^16^)		2657 (Ref. ^16^)	

The unit of the lattice parameter is Å. We also list the melting temperature of each material, which is given in K.

### First-principles calculation

To analyze electronic structures and optical properties, we performed ab initio density functional calculations for the four materials WC, W, TiN, and TiC selected in “TiCN-based cermet” by using Quantum Espresso package^17^. We used the Perdew–Burke–Ernzerhof type^18^ for the exchange-correlation functional, and the norm-conserving pseudopotentials are generated by the code ONCVPSP (Optimized Norm-Conserving Vanderbilt PSeudopotential)^19^ and are obtained from the PseudoDojo^20^. We used a 32 $\times$ 32 $\times$ 32 Monkhorst–Pack k-mesh for the Brillouin zone integration. The kinetic energy cutoff is set to be 96 Ry for the wave functions and 384 Ry for the charge density. The Fermi energy was estimated with the Gaussian smearing techniques with the width of 0.001 Ry^21^. The crystal structures were fully optimized, where the WC is an hcp structure, the W is a bcc structure, and the others TiC and TiN are an fcc structure. The resulting lattice parameters are listed in Table 1 and are in good agreement with the experimental results^11–13^.

Ab initio calculations for maximally localized Wannier function^22,23^ and optical properties were performed with RESPACK^24,25^. For the Wannier function analysis of the WC, TiC, and TiN compounds, we constructed the Wannier orbitals for the *s* and *p* orbitals of C and N, and the *d* orbitals of Ti and W, reproducing the original Kohn–Sham band structures in the energy range from − 18 to 10 eV with reference to the Fermi level. For the bulk W, we constructed the Wannier functions for W-*s*, W-*p*, and W-*d* orbitals. We performed a decomposition analysis for electronic density of states (DOS) with the resulting Wannier functions. Optical calculations were performed as follows: The energy cutoff for the dielectric function is set to 10 Ry. The total number of bands used in the polarization calculation is 36 for WC, 56 for W, 32 for TiC, and 34 for TiN, which covers unoccupied states up to $\sim$ 40 eV above the Fermi level. The integral over the Brillouin zone was calculated with the generalized tetrahedron technique^26^ with a smearing of 0.01 eV. For the WC, we performed band and optical calculations considering the spin-orbit coupling, but the obtained results hardly changed (see “Spin-orbit interaction effects in WC”)^27^. Therefore, in the present discussion, we basically analyze the results based on calculations that do not consider the spin-orbit coupling.

## Results and discussions

### Electronic structure

**Figure 3:**
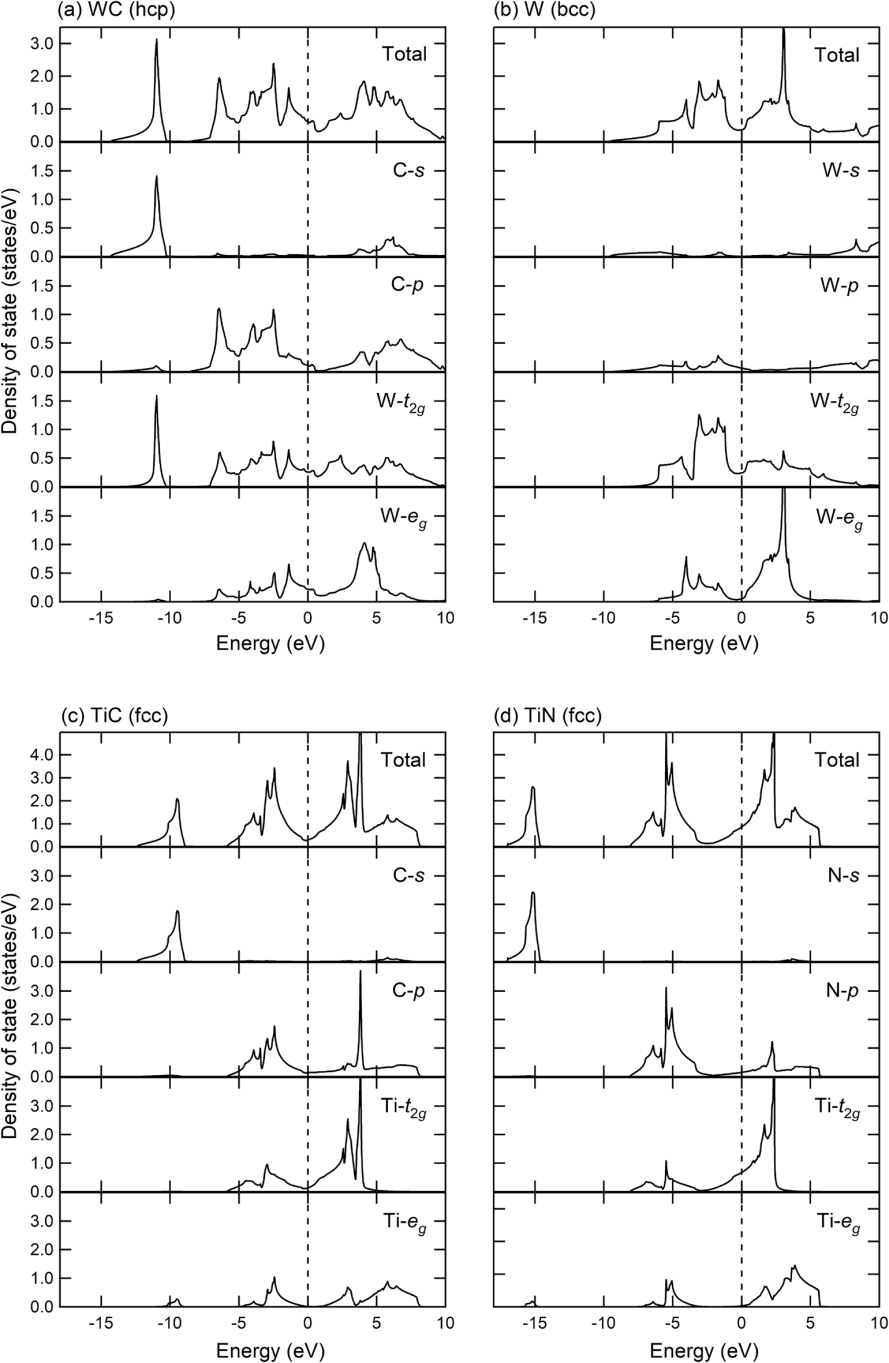
Calculated DOS of (**a**) WC, (**b**) W, (**c**) TiC, and (**d**) TiN, and the decomposition into each atomic contribution. We note that these calculations are performed with the maximally localized Wannier functions^22,23^. The energy zero is the Fermi level denoted by a dashed line. For the transition metals W and Ti, we further decompose the contribution into $t_{2g}$ and $e_g$ orbitals.

We show in Fig. 3 our calculated density functional DOS of WC (Fig. 3a), W (Fig. 3b), TiC (Fig. 3c), and TiN (Fig. 3d) and those decomposition into atomic contribution based on the Wannier function, where, for transition metals W and Ti, the local-*d* DOS is decomposed to the $t_{2g}$ and $e_g$ orbital contributions. For the WC, we see that the W-*d* and C-*p* orbitals are well hybridized, while in the TiN, the Ti-*d* and N-*p* orbitals are not strongly hybridized, indicating an enhancement of ionic character of the Ti–N bond. Basically, such a bonding tendency can be understand from the view of electronegativity of each atom; W (1.7), Ti (1.54), C (2.55), and N (3.04), taken from Ref. ^28^. Density-functional band-structure data of these materials are given in Supplementary Fig. S1.

### Optical property

**Figure 4:**
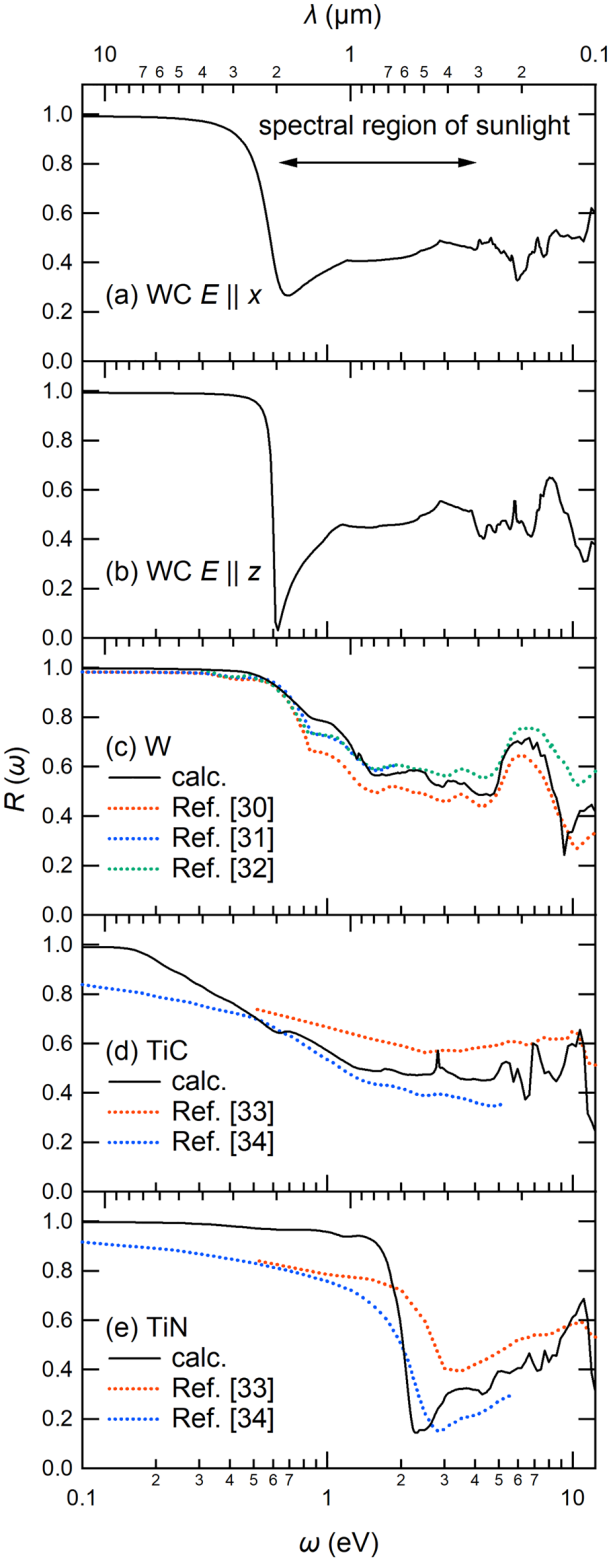
Calculated reflectivity spectra of (**a**) WC ($E\parallel x$), (**b**) WC ($E\parallel z$), (**c**) W, (**d**) TiC, and (**e**) TiN as a function of photon energy $\omega$ or photon wavelength $\lambda$ (upper scale). The theoretical curves are given by black solid curves and are compared with the experimental results (colored dotted curves) for W^30–32^, TiC^33,34^, and TiN^33,34^. The spectral region of sunlight (0.3–2.0 $\upmu$m or 0.6–4.1 eV) is indicated with a double arrow in the panel (**a**).

To study optical property of the four materials, we calculated their reflectivity spectra as

1 $$\begin{aligned} R(\omega ) = \Biggl | \frac{ 1-\sqrt{\varepsilon (\omega )} }{ 1+\sqrt{\varepsilon (\omega )} } \Biggr |^2, \end{aligned}$$

where $\varepsilon (\omega )$ is a dielectric function in the random phase approximation (RPA) based on the Lindhard formula^29^. Figure 4 compares calculated reflectivity spectra of the WC, W, TiC, and TiN. For the WC, we see a clear plasma edge near 0.6 eV (2 $\upmu$m) (Fig. 4a, b), and this energy just corresponds to the cutoff wavelength mentioned in Fig. 1b. For the TiN (Fig. 4e), we also see a sharp plasma edge, but its energy is rather high as 2 eV (0.6 $\upmu$m). For other compounds W and TiC, the reflectivity gradually decreases with the frequency $\omega$. We note that the theoretical reflectivity spectra are in a reasonable agreement with the experiment for the W^30–32^, TiC^33,34^ and TiN^33,34^. To our knowledge, however, there are no experimental data on the reflectivity spectrum of the WC with the hcp structure.

To understand details of the calculated reflectivity spectra, we perform a decomposed analysis for the dielectric function $\varepsilon (\omega )$ as

2 $$\begin{aligned} \varepsilon (\omega )=\varepsilon _{intra}(\omega )+\varepsilon _{inter}(\omega ), \end{aligned}$$

where $\varepsilon _{intra}(\omega )$ is the Drude term which is described as

3 $$\begin{aligned} \varepsilon _{intra}(\omega )=1-\frac{\omega _0}{\omega -i\delta }, \end{aligned}$$

with $\omega _0$ and $\delta$ being the bare plasma frequency and smearing factor, respectively^24^. The $\varepsilon _{intra}(\omega )$ describes the dielectric response due to the intraband excitation at the Fermi level, and the $\varepsilon _{inter}(\omega )$ represents the dielectric response involving the interband excitation. We now write the $\varepsilon (\omega )$ with introducing switching parameters *s* and *t* as

4 $$\begin{aligned} \varepsilon (\omega )= 1-s\frac{\omega _0}{\omega -i\delta }+t\varepsilon _{inter}(\omega ), \end{aligned}$$

where the $s=t=1$ case describes the full RPA dielectric function in Eq. (2). On the other hand, with the setting of ($s=1$, $t=0$), the dielectric function considers only the Drude term [(Eq. (3)], while the setting of ($s=0$, $t=1$) describes the dielectric function with only the interband excitation. By comparing the dielectric functions under the various parameter setting, we discuss details of the dielectric functions. In the practical calculations, by taking the inverse of the dielectric matrix in the plane-wave basis, we calculate the macroscopic dielectric functions with $\textbf{G}=\textbf{G}^{\prime }=\textbf{0}$ and the $\textbf{q}\rightarrow \textbf{0}$ limit^25^, where $\textbf{G}$ and $\textbf{G}^{\prime }$ are reciprocal lattice vectors, and $\textbf{q}$ is the wave vector in the Brillouin zone.

In the discussion of the dielectric function, we focus on the two quantity; one is the plasma frequency $\omega _p$ characterized as the zero point of the dielectric function, and the other is the plasmon-scattering strength $W_p$ due to the interband excitations, estimated from the imaginary part of the dielectric function at $\omega =\omega _p$. For better solar absorber, it is desirable that the $\omega _p$ is near the cutoff energy 0.6 eV (or the cutoff wavelength 2 $\upmu$m), and around there, the $W_p$ should be small.

**Figure 5:**
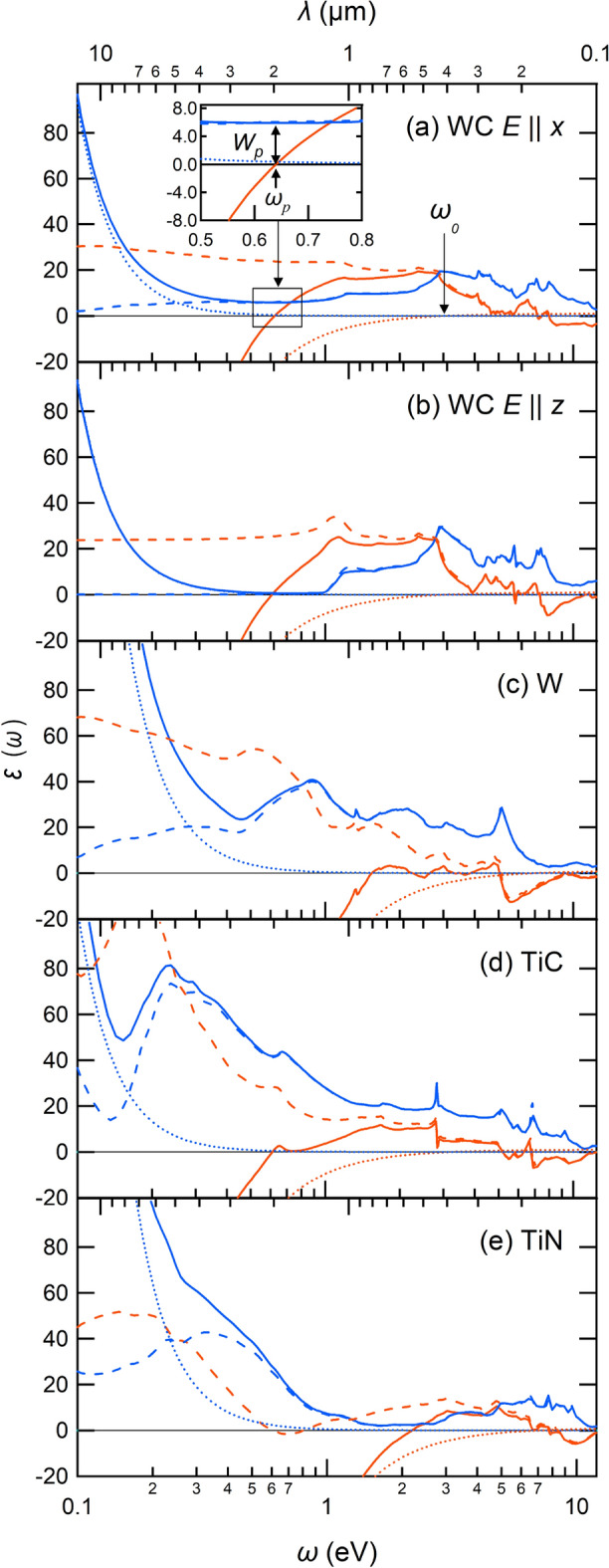
Calculated dielectric function of (**a**) WC ($E\parallel x$), (**b**) WC ($E\parallel z$), (**c**) W, (**d**) TiC, and (**e**) TiN. Red and blue solid curves describe the real and imaginary parts of the dielectric function in Eq. (2), respectively. Red and blue dotted curves represent the real and imaginary parts of the Drude dielectric function in Eq. (3), respectively. The dashed red and blue curves are the real and imaginary parts of the dielectric function without the Drude term [i.e., $s=0$ and $t=1$ in Eq. (4)], including only the contribution from the interband transitions. An inset in the panel (**a**) shows an enlarged view in the energy range [0.5 eV: 0.8 eV], in which the plasma frequency $\omega _p$ and plasmon scattering $W_p$ are indicated. The bare plasma frequency $\omega _0$ indicated by an arrow is specified from the Drude dielectric function.

Figure 5 compares ab initio dielectric functions of the four materials. The solid red and blue curves are the real and imaginary parts of the full macroscopic dielectric function [Eq. (2) or $s=t=1$ in Eq.(4)], respectively. The dotted red and blue curves respectively describe the real and imaginary parts of the macroscopic dielectric function only considering the Drude term [Eq. (3) or $s=1$ and $t=0$ in Eq. (4)]. The dashed red and blue curves respectively represent the real and imaginary parts of the macroscopic dielectric function with only considering the interband transitions [$s=0$ and $t=1$ in Eq. (4)]. Through the comparison, we find the several aspects, and let us consider the case of the WC as an example (Fig. 5a):

1. With neglecting the Drude contribution (dashed curves), the resulting dielectric function gives the insulating behavior; the real part of the dielectric function (the dashed red curve) gives the finite value at the limit $\omega \rightarrow 0$, and the imaginary part (the dashed blue curve) goes to zero of this limit. In the case of the WC, the real part is flat around the low-energy region.
2. By considering the Drude term (solid curves), the dielectric function (the solid red curve) rapidly drops toward minus infinite and therefore the zero point is formed in the low-energy region.
3. Thus, the bare plasma excitation $\omega _0$ ($\sim$ 3 eV) due to the $\varepsilon _{intra}(\omega )$, denoted by the arrow in Fig. 5a, is largely reduced to $\omega _p \sim$ 0.6 eV (the arrow in the inset) by considering the interband transition.
4. This trend is basically common among all the materials.
5. An interesting point is that, in the case of the WC (Fig. 5a,b), $W_p$ is appreciably small around the plasma excitation $\omega _p$. Thus, the sharp plasma edge appears in the reflectivity spectra of the WC in Fig. 4a,b.

### Fitting of simple models to ab initio spectra of WC

**Figure 6:**
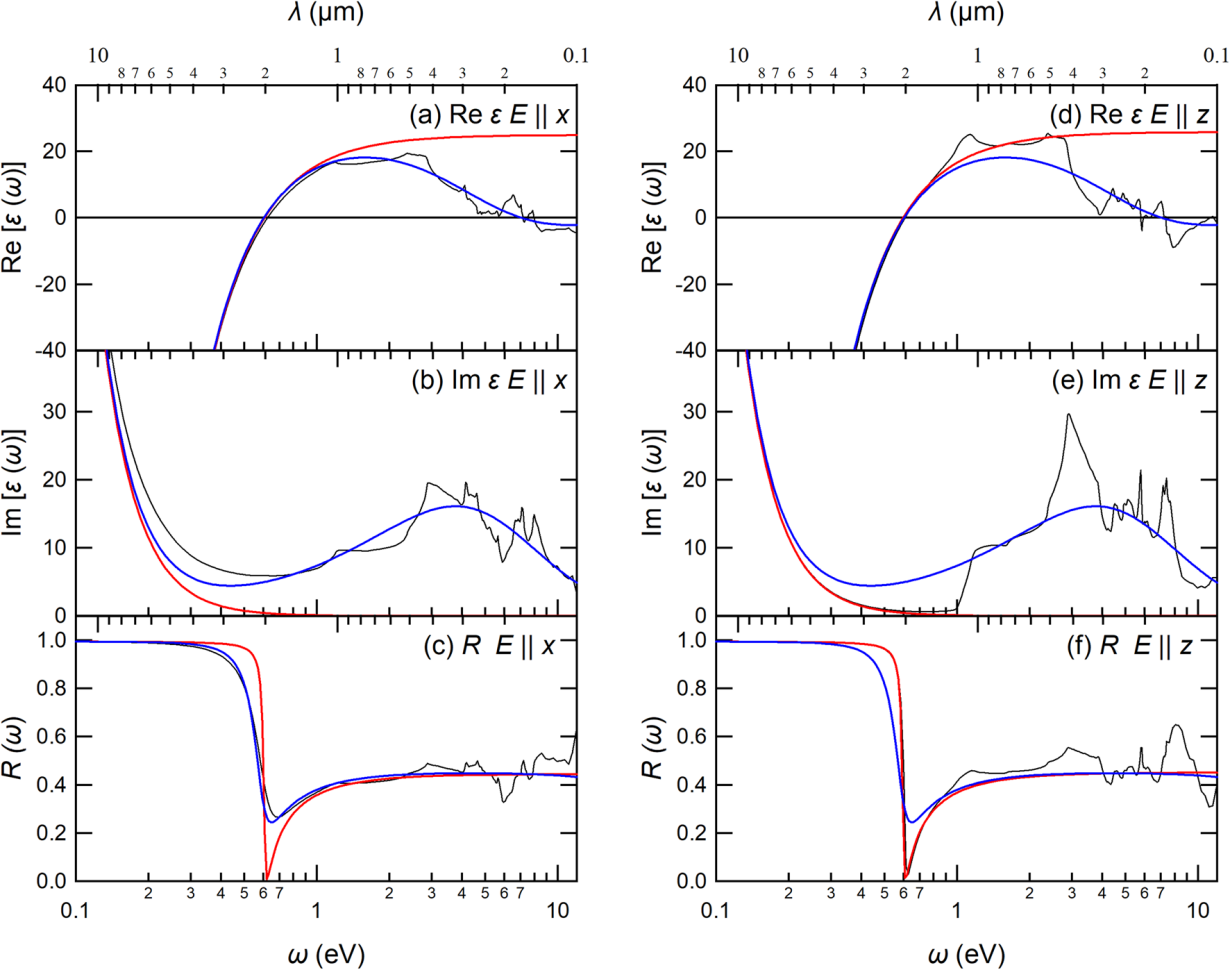
Comparison among ab initio results (black curves), DS model in Eq. (5) (red curves), and DL model in Eq. (6) (blue curves) of WC. Panels (**a–f**) show the results for $E \parallel x$ and $E \parallel z$, respectively. Also, the panels (**a,d**) show the real part of the dielectric function, (**b,e**) show the imaginary part of the dielectric function, and (**c,f**) describe the reflectance spectra.

In this subsection, for a deep understanding of the ab initio optical spectra of the WC, we consider simple models reproducing the ab initio data. The Drude-Sommerfeld (DS) model^35,36^ is given as

5 $$\begin{aligned} \varepsilon (\omega )=\varepsilon _{\infty } - \frac{\Omega _p^2}{\omega ^2+i\omega \Gamma }, \end{aligned}$$

where $\Omega _p$ is a model plasma frequency, $\Gamma$ is a linewidth, and $\varepsilon _{\infty }$ is a parameter due to interband response. On top of that, we consider the following Drude–Lorenz (DL) model^36^ as

6 $$\begin{aligned} \varepsilon (\omega )=\varepsilon _{\infty } - \frac{\Omega _p^2}{\omega ^2+i\omega \Gamma } + \sum _{i}^{M}\frac{\Omega _{pi}^2}{\Omega _i^2-\omega ^2-i\omega \Gamma _i}, \end{aligned}$$

where $\Omega _i$ is the *i*-th oscillator frequency, $\Omega _{pi}$ is the *i*-th model plasma frequency, and $\Gamma _i$ is the *i*-th linewidth. Also, *M* is the total number of oscillators. In the present study, we consider the *M* = 1 case for simplicity. We performed parameter fittings for these models using the software of Ref. ^36^. The resulting parameters are summarized in Table 2. Figure 6 compares the fitted DS model [Eq. (5)] (red curves), DL model [Eq. (6)] (blue curves), and the ab initio results (black curves) for the WC. The DS model is valid in the low-energy region near the plasma frequency, and the fitting of the DS model to ab initio data is performed for the $\omega$ region down to 1 eV. Therefore, the fitting of the DS model is not good in the high frequency region. This point is well improved by considering the individual excitation with the DL model. We can see a reasonable agreement between the DL model and ab initio results.

**Table 2:** Fitted parameters in the DS model in Eq. (5) and the DL model with $M=1$ in Eq. (6).

	DS		DL ($M=1$)	
	$E \parallel x$	$E \parallel z$	$E \parallel x$	$E \parallel z$
$\varepsilon _\infty$	24.949	25.842	0.936	1.526
$\Omega _p$	3.033	3.040	3.033	3.040
$\Gamma$	0.010	0.010	0.010	0.010
$\Omega _1$	–	–	6.596	5.438
$\Omega _{p1}$	–	–	32.933	27.649
$\Gamma _1$	–	–	13.337	8.223

The unit is given as eV except for $\varepsilon _{\infty }$.

### Temperature dependence of ab initio reflection spectra of WC

In this subsection, we consider a temperature dependence of the reflectance spectra of WC. Followed by Ref. ^37^, we evaluated the temperature dependence of the reflectance spectrum. In this approach, the lattice-expansion effect is considered; the lattice constants at given temperatures are estimated with the experimental thermal-expansion coefficient as

7 $$\begin{aligned} a(T)= & {} a_{293}\bigl [ 1+\rho _a (T-293) \bigr ], \end{aligned}$$

8 $$\begin{aligned} c(T)= & {} c_{293}\bigl [ 1+\rho _c (T-293) \bigr ], \end{aligned}$$

where *a*(*T*) and *c*(*T*) are the *a* and *c* parameters of WC at the temperature *T*, respectively. Also, $a_{293}$ and $c_{293}$ are the lattice constants at 293 K, and $\rho _a$ and $\rho _c$ are the thermal-expansion coefficients along the *a* and *c* direction, respectively. In the experiment^38^, the $a_{293}$, $c_{293}$, $\rho _a$, and $\rho _c$ are 2.907 (Å), 2.837 (Å), 5.2 $\times$ 10$^{-6}$ (K$^{-1}$), and 7.3 $\times$ 10$^{-6}$ (K$^{-1}$), respectively. Based on this treatment, we evaluated the lattice constants at the given temperatures, and performed ab initio band calculations for the resulting structures. In the optical calculations, we introduce a smearing parameter $\delta$ (Ref. ^25^). So, the optical calculations were performed with the smearing parameters of the corresponding temperatures.

Table 3 lists our estimated lattice parameters at given temperatures ranging from 300 and 1000 K. In principle, the lattice expansion seems to be small. For reference, we give the ab initio optimized lattice parameters with the use of exchange correlation functional of generalized gradient approximation (GGA). The GGA-level calculation is well known to give somewhat large lattice constants compared to the experimental lattice parameters. So, it is not surprising that the theoretical value is larger than the lattice parameter of 1000 K.

**Table 3:** Temperature dependence of lattice parameters estimated with the experimental thermal-expansion coefficient and the lattice structure at 293 K [Eqs. (7, 8)] (Ref.^38^).

*T* [K]	*a* [Å]	*c* [Å]	$\delta$ [eV]
300	2.907	2.837	0.026
500	2.910	2.841	0.043
700	2.913	2.845	0.060
1000	2.918	2.852	0.086
Ab initio opt.	2.922	2.847	0.010

For reference, we give the ab initio optimized lattice parameters within the GGA level. Also, $\delta$ is a smearing parameter introduced in the optical calculations, and the value is set to the corresponding temperature.

**Figure 7:**
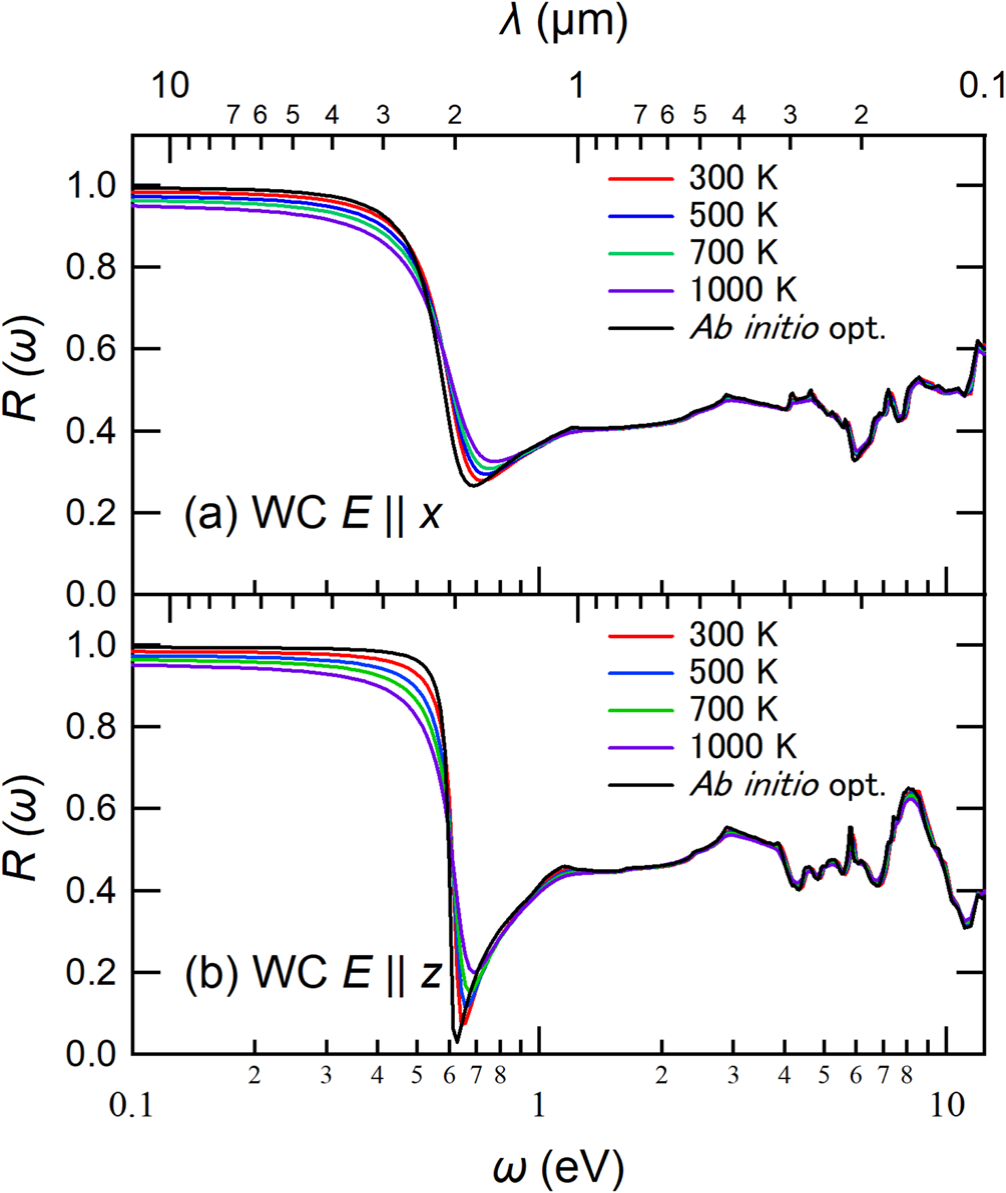
Temperature dependence of reflectivity of WC, where *T* = 300 K (red curves), 500 K (blue curves), 700 K (green curves), and 1000 K (purple curves) are displayed. For reference, the spectra with the *ab initio* optimized structure are also shown with black curves. The panels (**a,b**) show the results for $E \parallel x$ and $E \parallel z$, respectively.

Figure 7 shows our calculated temperature dependence of the reflectance spectra of WC, where we compare the spectra of *T* = 300 K (red curves), 500 K (blue curves), 700 K (green curves), 1000 K (purple curves), and ab initio optimized structure (Black curves). We can see the moderate temperature dependence of the spectrum; the plasma-edge position is basically unchanged but the shape becomes broader. We note that the resulting spectral change are basically due to the change in the smearing parameters. Ab initio computational approaches to consider the temperature effect on the electronic states more seriously have also been proposed^39,40^, which is an important future work.

### Spin–orbit interaction effects in WC

**Figure 8:**
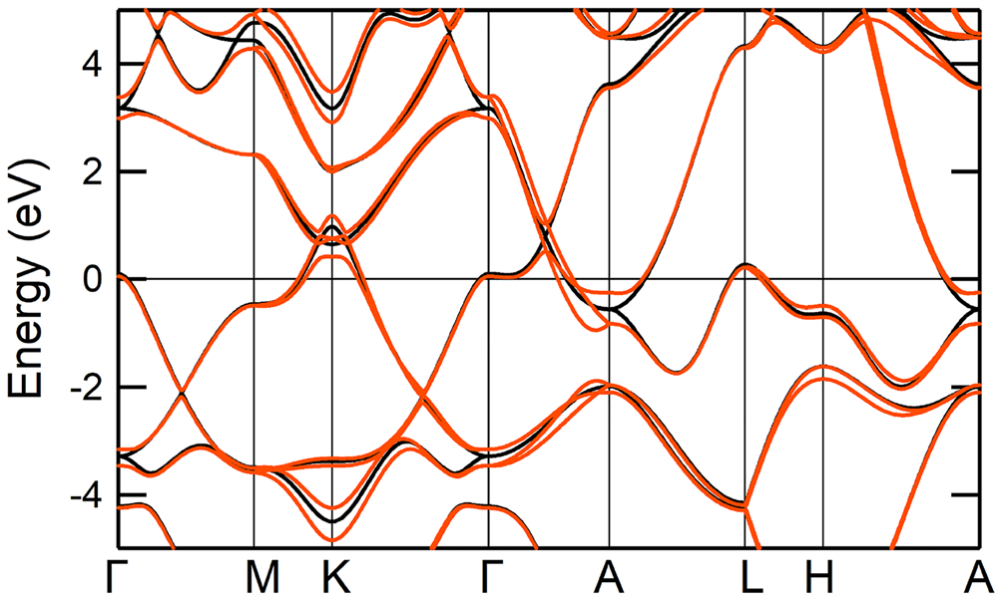
Comparison between ab initio density functional band structure of WC with (red solid curves) and without (black solid curves) the SOI. The energy zero is the Fermi level. Dispersions of the WC are plotted along the high symmetry points in the Brillouin zone, where $\Gamma$ = (0, 0, 0), M = (1/2, 0, 0), K = (1/3, 1/3, 0), A = (0, 0, 1/2), L = (−1/2, 0, 1/2), and H = (1/3, 1/3, 1/2), where the coordinates are represented in terms of basic vectors of the reciprocal lattice of the hcp lattice.

**Figure 9:**
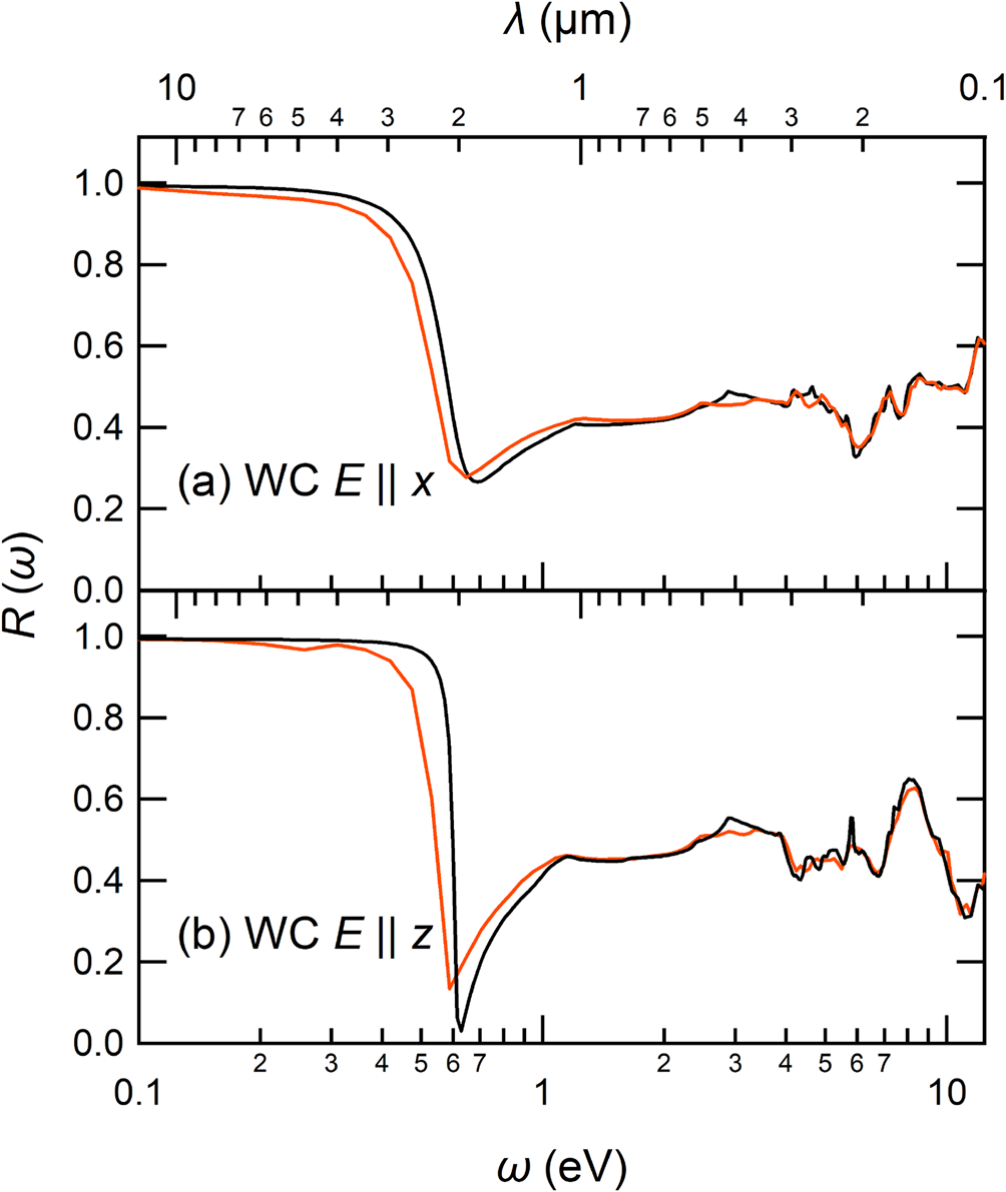
Comparison between ab initio reflectivity spectra of WC with (red solid curves) and without (black solid curves) the SOI. The panels (**a,b**) show the results of $E\parallel x$ and $E\parallel z$, respectively. The view of the figure is the same as Fig. 4.

Here, we discuss a spin–orbit interaction (SOI) effect in WC. The SOI of tungsten is known to be nearly 0.4 eV^41^. Figure 8 compares our calculated band structures with (red solid curves) and without (black solid curves) the SOI. The SOI can bring about a splitting of the low-energy bands, but the effect is basically small overall. We also compare in Fig. 9 our calculated reflectivity spectra, where the red and black solid curves are the results with and without the SOI, respectively. The spectra with the SOI are calculated with the spinor version of RESPACK^42^. We again see a small difference between the two results, so we think that the SOI effect can be ignored within the purpose of evaluating the reflectivity or absorptivity performance of the WC.

### Figure of merit for photothermal conversion

Solar absorptivity and thermal emissivity are widely used to evaluate the performance of solar selective absorbers. The solar absorptivity $\alpha _s$ is defined via the wavelength integral as^1,4,43^

9 $$\begin{aligned} \alpha _s = \frac{\int _{\lambda _l}^{\lambda _h} \bigl (1-R(\lambda )\bigr ) I_{sol}(\lambda ) d\lambda }{ \int _{\lambda _l}^{\lambda _h} I_{sol}(\lambda ) d\lambda }, \end{aligned}$$

where $R(\lambda )$ is the reflectivity spectra as a function the wavelength $\lambda$, which is taken from the present ab initio calculations. $I_{sol}(\lambda )$ is the spectral solar radiance (air mass of 1.5) taken from Ref. ^44^. The $\lambda _l$ and $\lambda _h$ are the lower and higher cutoff wavelengths, respectively, and were set to 0.28 $\mu$m and 4 $\mu$m in the present study. Similarly, the thermal emissivity at a temperature *T* is defined as follows^1,4,43^:

10 $$\begin{aligned} \varepsilon _t(T) = \frac{\int _{\lambda _L}^{\lambda _H} \bigl ( 1-R(\lambda ) \bigr ) I_{b}(T, \lambda ) d\lambda }{\int _{\lambda _L}^{\lambda _H} I_{b}(T, \lambda ) d\lambda }. \end{aligned}$$

Here $I_b (T,\lambda )$ is the spectral blackbody radiative intensity, which is taken from Ref. ^45^. The $\lambda _L$ and $\lambda _H$ are the lower and higher cutoff wavelengths for the emittance evaluation, respectively, and were set to 0.1 $\mu$m and 124 $\mu$m in the present study.

The usable heat $Q_H$ can be defined in terms of a heat generated from light absorption and a loss due to radiation as^43^

11 $$\begin{aligned} Q_H = B \alpha _s c I_0 - \varepsilon _t \sigma T^4, \end{aligned}$$

where $\sigma$, *c*, and $I_0$ are the Stefan–Boltzmann constant, the solar concentration, and the solar flux intensity, respectively. *B* is related to the transmittance of glass envelope, and is typically chosen to be 0.91 (Ref. ^3^). The first term of the right-hand side in Eq. (11) is the heat stored inside a material due to the light absorption and the second term describes the heat loss due to the material emmitance. The photothermal conversion efficiency $\eta _{\textrm{FOM}}$ of the solar absorber, called figure of merit (FOM)^43^, can be defined by dividing the above usable heat $Q_H$ by the incident solar energy as

12 $$\begin{aligned} \eta _{\textrm{FOM}} = \frac{Q_H}{cI_0} = \frac{B \alpha _s c I_0 - \varepsilon _t \sigma T^4}{cI_0} = B\alpha _s - \frac{\varepsilon _t \sigma T^4}{cI_0}. \end{aligned}$$

In the present calculation, we set *T* to 673 K, *c* to 80 suns, and $I_0$ to 1 kW/m$^2$. These are a standard condition^46^. We note that, in an ideal blackbody, i.e., $\alpha _s=\varepsilon _t=1$, the present condition of *T* = 673 K gives $B-\frac{\sigma T^4}{c I_0} \sim$ 0.76.

We summarize in Table 4 our calculated parameters of the present materials, characterizing the performance of the solar absorber. About the plasma frequency $\omega _p$, the WC and TiC are clearly small as 0.6 eV, but the WC plasmon scattering $W_p$ at $\omega =\omega _p$ is appreciably small as 0.7–5.9 compared to the TiC (41.8). Thus, the solar absorptivity $\alpha _s$ of the WC becomes appreciably high compared to the other materials, and yields a better performance of the figure of merit $\eta _{\textrm{FOM}}$. On the other hand, we comment that the $\alpha _s$ of WC is still not so high as 0.53–0.57, which can be improved with better choices of anti-reflection and/or infrared reflective layers sandwiching the cermet layer (Fig. 1), which is left as an important issue for the future study. While the FOM of the present study is lower than that of artificial solar selective absorbing coatings (SSACs), it is important to note that advanced SSACs with multi-layers can incorporate nano-particles at carefully controlled concentrations based on intricate optical designs. These artificial features address the weaknesses of SSACs mentioned in the introduction. On the other hand, the TiCN-based cermet serves as an intrinsic absorber. While pyromark 2500 is widely used as a black-body paint in concentrating solar power plants, its durability at high temperatures remains an ongoing issue^47^. This durability concern also applies to SSACs. However, the TiCN-based cermet which is basically a cutting device and is durable at high temperatures offers higher durability at lower costs compared to state-of-the-art SSACs.

**Table 4:** Summary of the bare plasma frequency $\omega _0$, plasma frequency $\omega _p$, the imaginary part of the dielectric function $W_p$ at $\omega =\omega _p$, solar absorptivity $\alpha _s$ in Eq. (9), thermal emissivity $\varepsilon _t$ at $T=673$ K in Eq. (10), and figure of merit $\eta _{\textrm{FOM}}$ for photothermal conversion in Eq. (12) at $T=673$ K.

Material	$\omega _0$	$\omega _p$	$W_p$	$\alpha _s$	$\varepsilon _t$	$\eta _{\textrm{FOM}}$
WC ($E\parallel x$)	3.03	0.63	5.90	0.57	0.04	0.51
WC ($E\parallel z$)	3.04	0.62	0.67	0.53	0.02	0.48
W	7.15	1.53	23.9	0.33	0.01	0.30
TiC	3.25	0.61	41.8	0.49	0.09	0.43
TiN	7.15	2.18	2.34	0.37	0.01	0.33
blackbody	–	–	–	1.00	1.00	0.76
SSACs (Ref. ^48^)	–	–	–	0.88–0.95	0.02–0.17	0.78–0.86

The $\omega _0$ and $\omega _p$ are given in the unit of eV. For reference, we list $\eta _{\textrm{FOM}}$ of an ideal balckbody ($\alpha _s=\varepsilon _t=1$), as well as artificial solar selective absorbing coatings (SSACs) taken from Table 2 of Ref. ^48^.

## Conclusion

In the present paper, we have studied electronic structures and optical properties of WC, W, TiC, and TiN, identified as major components in the TiCN-based cermet. We have found that the WC exhibits a sharp plasma edge due to the low-energy plasma excitation $\sim$ 0.6 eV (2 $\upmu$m), which just corresponds to a cutoff wavelength suitable for the solar selective absorber. We have checked that this result is unchanged with taking into account the SOI of W. We have analyzed the origin of the low-energy plasma edge and found that, in the WC, the plasmon scattering due to the interband transitions is strongly suppressed around the plasma excitation. This aspect directly reflects to the solar absorptivity, bringing about the better performance of the figure of merit for photothermal conversion. The solar absorptivity of the WC would further be improved to suppress the reflection due to the interband excitation with the fine-structure processing and/or introduction of reflection layers.

## Supplementary Information

Supplementary Information.

## Data Availability

The data that support the findings of this study are available from the corresponding author upon reasonable request.
