# Supplementary material for: Ab initio calculation for electronic structure and optical property of tungsten carbide in a TiCN-based cermet for solar thermal applications
Source: Sci Rep. 2023 Jun 9;13:9407. doi: 10.1038/s41598-023-36337-4 (PMC10256811; doi:10.1038/s41598-023-36337-4)
Supplement: Supplementary file 1 — Supplementary Information. [file 41598_2023_36337_MOESM1_ESM.pdf]

Supplemental Information for *Ab initio* calculation for electronic structure and optical property of tungsten carbide in a TiCN-based cermet for solar thermal applications

Shota Hayakawa[1], Toshiharu Chono[1], Kosuke Watanabe[2], Shoya Kawano[1,2], Kazuma Nakamura[1,2], and Koji Miyazaki[1,2,3]

[1]*Graduate School of Engineering, Kyushu Institute of Technology, Kitakyushu, 804-8550, Fukuoka, Japan*
[2]*Integrated Research Center for Energy and Environment Advanced Technology, Kyushu Institute of Technology, Kitakyushu, 804-8550, Fukuoka, Japan*
[3]*Graduate School of Engineering, Kyushu University, Fukuoka, 819-0395, Fukuoka, Japan*

As supplemental material of the present paper, we attach density-functional band-structure data: Figure S1 compares our calculated band structures of WC (Fig. S1a), W (Fig. S1b), TiC (Fig. S1c) , and TiN (Fig. S1d), where we see that all the materials are metal. Our results are in good agreements with the previous density functional calculations [1, 2, 3].

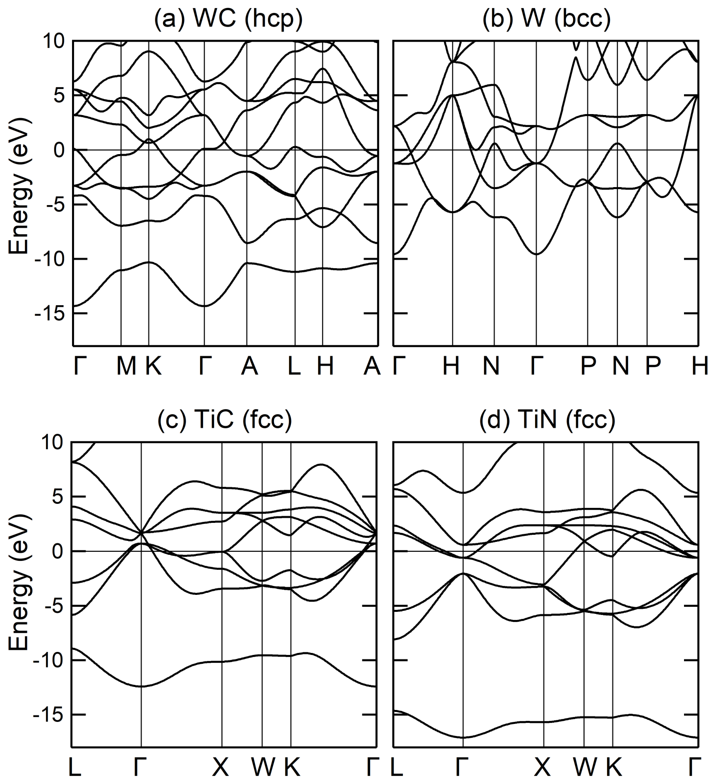

Figure S1: *Ab initio* density functional band structure of (a) WC, (b) W, (c) TiC, and (d) TiN. The energy zero is the Fermi level. Dispersions of the WC are plotted along the high symmetry points in the Brillouin zone, where Γ= (0, 0, 0), M = (1/2, 0, 0), K = (1/3, 1/3, 0), A = (0, 0, 1/2), L = (−1/2, 0, 1/2), and H = (1/3, 1/3, 1/2), where the coordinates are represented in terms of basic vectors of the reciprocal lattice of the hcp lattice. In the band dispersion of the W, H = (1/2, −1/2, 1/2), N = (0, 0, 1/2), P = (1/4, 1/4, 1/4), and these coordinates are represented in the basic vectors of the bcc reciprocal lattice. Finally, in the band dispersions of TiC and TiN, L = (0, 1/2, 1/2), X = (1/2, 0, 1/2), W = (1/2, 1/4, 3/4), K = (3/8, 3/8, 3/4), which are based on the basic vectors of the fcc reciprocal lattice.

References

[1] Dmitrii V. Suetin, Igor R. Shein, and Alexander L. Ivanovskii. Structural, electronic properties and stability of tungsten mono- and semi-carbides: A first principles investigation. *Journal of Physics and Chemistry of Solids*, 70(1):64–71, 2009.

[2] N. Egede Christensen and B. Feuerbacher. Volume and surface photoemission from tungsten. i. calculation of band structure and emission spectra. *Phys. Rev. B*, 10:2349–2372, Sep 1974.

[3] R. Ahuja, O. Eriksson, J. M. Wills, and B. Johansson. Structural, elastic, and high-pressure properties of cubic tic, tin, and tio. *Phys. Rev. B*, 53:3072–3079, Feb 1996.
